# Multicolor-tunable room-temperature afterglow and circularly polarized luminescence in chirality-induced coordination assemblies†

**DOI:** 10.1039/d2sc05353e

**Published:** 2022-11-07

**Authors:** Hui Liu, Dan-Dan Ren, Peng-Fu Gao, Kun Zhang, Ya-Pan Wu, Hong-Ru Fu, Lu-Fang Ma

**Affiliations:** College of Chemistry and Chemical Engineering Luoyang Normal University Luoyang 471934 P. R. China hongrufu2015@163.com mazhuxp@126.com; College of Materials and Chemical Engineering China Three Gorges University Yichang 443002 P. R. China; College of Chemistry and Chemical Engineering Henan Polytechnic University Jiaozuo 454003 P. R. China

## Abstract

Dynamic long-lived multicolor room temperature afterglow and circularly polarized luminescence (CPL) are promising for optoelectronic applications, but integration of these functions into a single-phase chiroptical material is still a difficult and meaningful challenge. Here, a nitrogen-doped benzimidazole molecule 1*H*-1,2,3-triazolopyridine (Trzpy) showing pure organic room-temperature phosphorescence (RTP) acted as a linker, and then, we propose a chirality-induced coordination assembly strategy to prepare homochiral crystal materials. Two homochiral coordination polymers DCF-10 and LCF-10 not only exhibit multicolor-tunable RTP, the color changed from green to orange under various excitation wavelengths, but also show remarkable excitation-dependent circularly polarized luminescence (CPL), and the dissymmetry factors of CPL in DCF-10 and LCF-10 are 1.8 × 10^−3^ and 2.4 × 10^−3^, respectively. Experimental and theoretical studies demonstrated that molecular atmospheres with different aggregation degrees give rise to multiple emission centers for the generation of multicolor-tunable emission. The multicolor-tunable photophysical properties endowed LCF-10 with a huge advantage for multi-level anti-counterfeiting. This work opens up new perspectives for the development and application of color-tunable RTP and CPL.

## Abstract 2

A pair of chiral coordination polymers were synthesized *via* reticular chemistry, exhibiting multicolor-tunable RTP and excitation-dependent CPL, and showing the multiple anti-counterfeiting. It provides a new way to prepare chiral optical materials.

## Graphical abstract

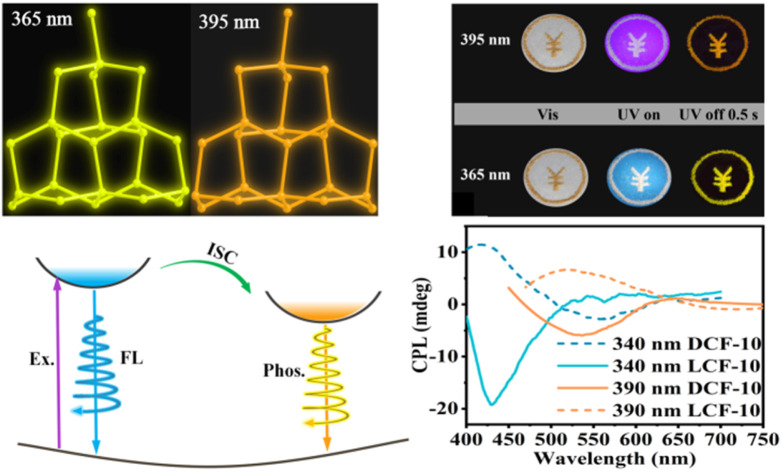

## Introduction

Organic room-temperature phosphorescence (ORTP) has drawn broader attention owing to its rich excited state and long-lived triplet manifold, showing potential applications in the fields of photoelectric display,^1^ logical encryption,^2^ and biological probes.^3^ However, due to the poor spin–orbit coupling (SOC), ultrafast triplet exciton deactivation, and susceptibility of triplet excitons, achieving efficient room-temperature phosphorescence is difficult.^4–6^ To date, many efforts have been made; in total, it can be summarized into two parts, introducing heteroatoms,^7^ halogen atoms,^8,9^*etc.* to promote the intersystem crossing (ISC) and providing a robust environment to suppress the non-radiative decay, such as crystal engineering^10,11^ and guest-doping into host matrices.^12–15^

Recently, ORTP entered a new development stage, namely, dynamic RTP. Compared to static RTP, the phosphorescence behaviors including emission range, lifetime, and emission type, can generate a dynamic response, such as light-, heating-, pH-, or mechanical-responsive RTP.^16–18^ Significantly, benefiting from the sensitive response, sophisticated information, and smart reversibility, dynamic RTP presents much wider prospects in practical applications. Excitation-dependent RTP, as a subclass of dynamic RTP, featuring color-tunable properties and multiple emission, has gained special attention. For instance, Huang's group reported a series of organic molecules with triazine as the core, showing excitation-dependent phosphorescence from blue to green when excited under UV illumination ranging from 250 to 390 nm.^19^ Zhao and coworkers reported multi-component amorphous polymers featuring color-tunable RTP. The multicolor long-lived luminescence was realized from blue to yellow by conjugating multiple organic phosphorescence emitting centers onto a polymer backbone.^20^ Although some achievements have been made, this kind of material is limited, and it is still a meaningful challenge to obtain excitation-dependent RTP with multicolor emission, to further explore the luminescence mechanism.

Circularly polarized luminescence (CPL) can efficiently reflect the excited state of a chiral structure, and circularly polarized phosphorescence (CPP)-active materials, which integrate the chirality and triplet excited state characteristics, have generated much interest in advanced application in 3D displays^21,22^ and chiroptical devices.^23,24^ Although great developments have been made in CPP-active materials by chiral molecular engineering,^25,26^ co-crystallization,^27,28^ and supramolecular self-assembly,^29,30^ it is difficult to tune and balance the chirality transfer and energy transfer between the chiral moieties and the RTP chromophores under ambient conditions, and CPL-active room-temperature phosphorescence materials exhibiting dynamic multicolor emission in single-phase systems are very rare.

Here, through comprehensive consideration of molecular configuration, the luminescence mechanism, and the generation and transfer of chirality, we propose an efficient strategy to realize color-tunable luminescence from a small molecule 1*H*-1,2,3-triazolo[4,5-*b*]pyridine (Trzpy). Firstly, as a benzimidazole derivative, the nitrogen-rich structure can effectively promote the spin–orbit coupling, further improving the intersystem crossing (ISC) from singlet-to-triplet excited states.^31^ Secondly, Trzpy molecules maybe form molecular atmospheres with different aggregation degrees through hydrogen bonds and π–π interactions, such as 1,3,5-triazinane-2,4,6-trione, and both are nitrogen-rich azole molecules.^32^ Beyond that, the Trzpy molecule can act as a linker; the chiral coordination polymers can be constructed with the introduction of the enantiopure organic building blocks *via* chiral reticular chemistry, and then, circularly polarized luminescence could be obtained.

As expected, the long lifetime room-temperature phosphorescence was obtained from Trzpy crystalline powder. Significantly, a pair of dia-type homochiral coordination polymers DCF-10 and LCF-10 (DCF = d-configuration chiral ligand-based chiral framework) were prepared through the self-assembly of Trzpy and d/l-alanine, simultaneously exhibiting excitation-dependent room-temperature phosphorescence and circularly polarized afterglow. The color of dynamic afterglow emission changed from green to orange under various excitation wavelengths. We have demonstrated that these coordination polymers have great potential in multicolor anti-counterfeiting application.

## Results and discussion

### Materials preparation and photophysical properties

**Fig. 1:**
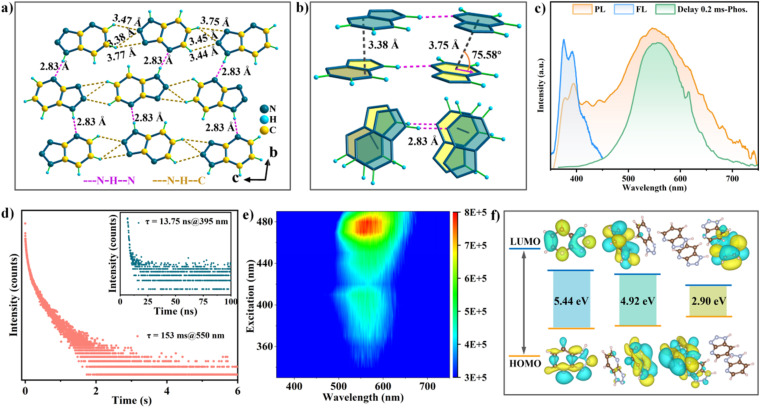
Photoluminescence properties of Trzpy crystal powder under ambient conditions. (a) Molecular packing and intermolecular interaction of the Trzpy single crystal in the direction of the *a*-axis; (b) molecular stacking patterns of Trzpy showing the side view and top view, respectively; (c) the photoluminescence (PL), fluorescence (FL), and delayed phosphorescence spectra of Trzpy crystal powder (the delay time is 0.2 ms); (d) steady-state phosphorescence spectra of Trzpy crystal powder (red line) at 550 nm with a delay time of 0.1 ms under ambient conditions. The inset shows the fluorescence lifetime profile for Trzpy crystal powder (dark green line); (e) excitation–phosphorescence mapping of Trzpy crystal powder at room temperature; (f) molecular orbitals for the Trzpy monomer, dimer linked by hydrogen bonds, and tetramer coupled by hydrogen bonds and π⋯π bonds.

The luminescence behaviors of Trzpy crystalline powder were investigated under ambient conditions. After a preliminary attempt, the naked-eye green afterglow lasting approximately 2.0 s can be observed after turning off the 365 nm UV lamp. Meanwhile, the solid-state UV-visible (UV-vis) absorbance and the excitation spectra were recorded. The UV-vis curve covers a narrow region in the range of 255–296 nm (Fig. S1†), which was mainly assigned to π–π* and n–π* transitions in the conjugated aromatic ring. The prompt photoluminescence (PL) of Trzpy exhibits a single blue emission at 395 nm with a lifetime of 13.75 ns under 260 nm excitation at room temperature (Fig. 1d), which is assigned to the fluorescence of Trzpy. The delayed PL spectra were also monitored. With a delay time of 0.1 ms, one peak was still observed at 550 nm (Fig. 1c). The steady-state luminescence spectrum of Trzpy was also recorded, and the emission intensity covering the whole emission was gradually decreased with varying the temperature from 73 to 298 K (Fig. S13†). Meanwhile, the time-resolved decay spectra were recorded, and the delayed lifetimes of afterglow at 550 nm are about 206.21 and 110.33 ms at 77 and 393 K, respectively, confirming that the afterglow at 550 nm belongs to a persistent phosphorescence. The total Commission International de l'Eclairage (CIE) coordinates of photoluminescence (PL) of Trzpy are (0.35, 0.40), and the CIE coordinates of fluorescence and phosphorescence are (0.17, 0.20) and (0.40, 0.52), respectively (Fig. S14†).

To more clearly figure out the afterglow properties of the different aggregates of Trzpy molecules, the photophysical properties were assessed in water and organic solvents at μM concentrations. Interestingly, Trzpy molecules could completely dissolve in cold water forming a 1 × 10^−5^ M solution, and two weak peaks were observed at 200 and 285 nm, respectively. When the concentration was increased to 1 × 10^−3^ M, even 1 × 10^−2^ M, although the intensity becomes stronger, the zone of absorption peaks has almost no bathochromic shift (Fig. S6–S12†). A similar situation was observed in other solvents, such as chloroform, dioxane, ethanol, and DMF; meanwhile, the absorption bands are nearly unchanged along with the variation of solvent polarity. This indicates that no intermolecular charge transfer occurs in aggregates.^33,34^ It should be pointed out that no phosphorescence emission was detected by the delayed photoluminescence spectra, except in 1 × 10^−2^ M chloroform (Fig. S15†). This is because Trzpy has poor solubility in such high concentration chloroform solution, showing the cluster-type aggregates. Importantly, the phosphorescence peak overlaps nearly perfectly with the long-wavelength region of the Trzpy crystalline sample. These results reveal that the phosphorescence emission of Trzpy significantly depends on its aggregated state.^35,36^

To gain a deeper insight into the dynamic luminescent behaviors of Trzpy molecules, we obtained a single crystal and revealed the molecular conformations and aggregation formations. Trzpy molecules form chains through N–H⋯N hydrogen bonds (2.83 Å) (Fig. 1a), and the chains further stack into a layer *via* the partial face to face stacking pattern with the moderate distance (3.38 Å) between the two parallel chains, where the overlap degree and intersection angle of the neighboring Trzpy molecules are approximately 64.3% and 75.58°, respectively. As shown in Fig. 1a, the distance of C–H⋯N ranges from 3.44 to 3.77 Å (Fig. 1a), suggesting that the layers finally form a three-dimensional supramolecular structure *via* the intermolecular interactions. Thus, through the packing arrangement, it can be observed that the layers formed by N–H⋯N hydrogen bonds and intermolecular interactions present a H-aggregation fashion through the intensive π⋯π interactions (3.38 Å) (Fig. 1b). Such a structure can supply a rigid environment, strongly restrain molecular vibration and disorder, maintain triplet excited state, and further endow the long-lived afterglow. Besides, three basic aggregate units can be confirmed: monomer, dimer linked by hydrogen bonds, and tetramer coupled by hydrogen bonds and π⋯π bonds (Fig. 1b).

To investigate the long-lived phosphorescence emission, the theoretical analysis on the different aggregate states extracted from the single crystal was carried out. First, the highest occupied molecular orbital (HOMO) and the lowest unoccupied molecular orbital (LUMO) were calculated. As presented in Fig. 1f, in the dimer and tetramer, the electron clouds of the HOMO and LUMO spread on the isolated side, while the energy gap becomes smaller, 5.44 eV for the monomer and 2.90 eV for the tetramer.

Compared to the orbital energy level of the dimer, the energy gap of the tetramer dramatically reduced, indicating that hydrogen bonds can greatly enhance electron communication and the conjugation effect between molecules in the tetramer.^37^ Besides, the splitting energy (Δ*E*_ST_) between the lowest singlet energy levels (S_1_) and the lowest triplet energy levels (T_1_) of both monomer molecules (0.79 eV) and dimers (0.74 eV) is very close, but far higher than that in the tetramer (0.65 eV) (Fig. S16†), demonstrating that the H-aggregation pattern can efficiently maintain the highly active triplet and weaken the and non-radiative rates for ultralong phosphorescence.^38,39^ As a result, the well-organized aggregated state can significantly promote the phosphorescence emission.

**Fig. 2:**
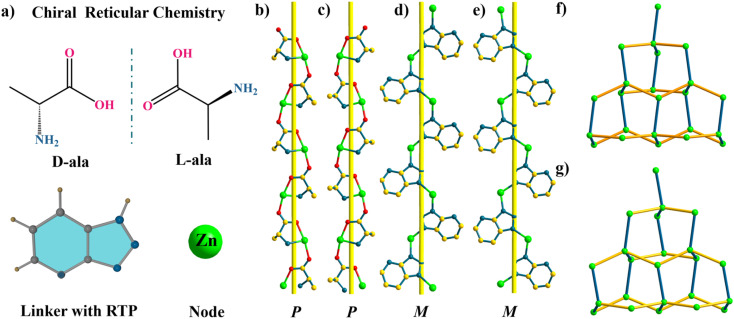
The construction strategy and single-crystal structure of homochiral coordination polymers. (a) The chiral ligands and organic luminophores; (b) d-ala coordinates with Zn^2+^ ions to form a P-type chain in DCF-10; (c) l-ala coordinates with Zn^2+^ ions to form a P-type chain in LCF-10; (d) M-type helical chains consist of Trzpy molecules in DCF-10; (e) M-type helical chains consist of Trzpy molecules in LCF-10; (f) the (4,4)-connected 3D network of DCF-10 with dia-topology; (g) the (4,4)-connected 3D network of LCF-10 with dia-topology.

Trzpy itself can serve as a ligand, and we try to prepare homochiral coordination polymers *via* reticular chemistry. A pair of enantiopure compounds were synthesized through the organization of Trzpy, d/l-alanine, and Zn(OAC)_2_·2H_2_O. Single-crystal X-ray analysis revealed that DCF-10 crystallizes in the orthorhombic system with chiral space group *P*2_1_2_1_2_1_. The base coordination unit consists of one Zn^2+^ ion, one deprotonated d-ala, and one deprotonated Trzpy. Each Zn^2+^ ion adopts a distorted trigonal-bipyramidal geometry, where two nitrogen atoms from two Trzpy and one nitrogen atom from d-ala form a triangular plane, and two axial positions are occupied by two oxygen atoms from two d-ala (Fig. S17†). The self-organization of d-ala anions and Zn^2+^ ions generates a one-dimensional P-type helical chain [Zn(d-ala)]_*n*_ (Fig. 2b). Interestingly, Trzpy molecules coordinate with Zn^2+^ ions to form a M-type helical chain [Zn(Trzpy)]_*n*_ (Fig. 2d). The P-type helical chains and the M-type helical chains further orthogonally connect each other *via* Zn^2+^ nodes to construct a three-dimensional framework. d-Ala and Trzpy can act as μ_2_-linkers, then, each Zn center can be regarded as a 4-connected node, and the whole framework can be shown as a dia-type network (Fig. 2f). It is necessary to mention that LCF-10 also crystallized in the orthorhombic *P*2_1_2_1_2_1_ space group; likewise, a M-type helical chain is observed in LCF-10 formed by l-ala ligands bridging Zn^2+^ ions. Notably, these two compounds are dense without any free pores.

**Fig. 3:**
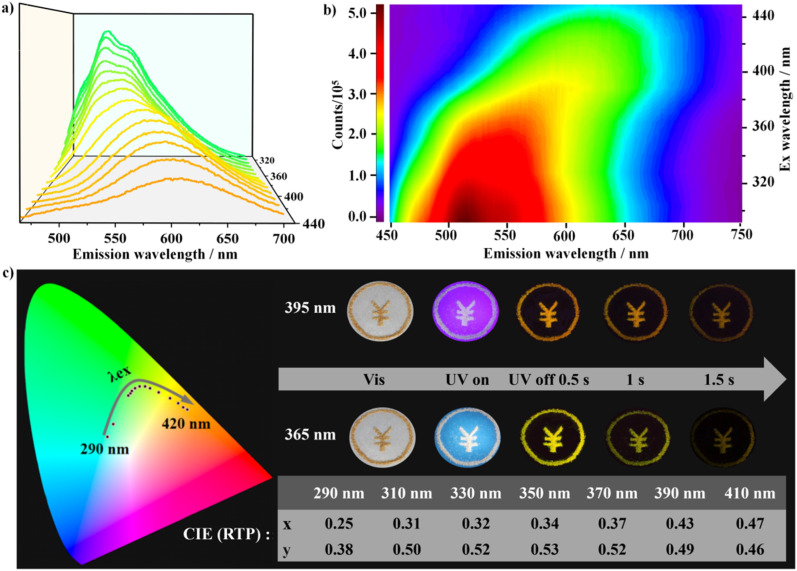
Photoluminescence properties of the DCF-10 crystal under ambient conditions. (a) The phosphorescence spectrum of the DCF-10 crystal under various excitation wavelengths from 310 to 420 nm; (b) excitation–phosphorescence emission mapping of the DCF-10 crystal; (c) excitation wavelength-dependent phosphorescence spectra and CIE coordinates of the DCF-10 crystal, inset photographs show the color change of the long-lasting phosphorescence under 365 and 395 nm excitation light sources, respectively.

**Fig. 4:**
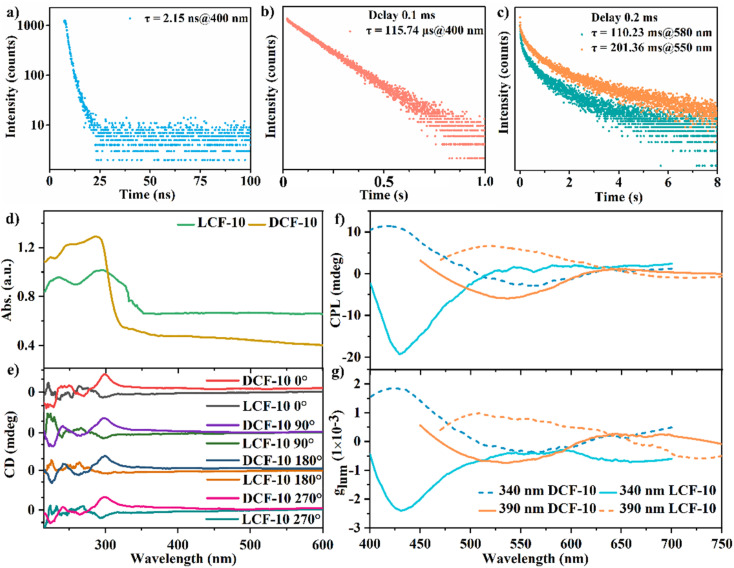
Photophysical properties of DCF-10 and LCF-10 under ambient conditions. (a) The normal fluorescence lifetime of DCF-10 crystal powder upon excitation at 340 nm; (b) the TTA-generated delayed fluorescence lifetime of DCF-10 crystal powder upon excitation at 340 nm; (c) the time-resolved decay curves of DCF-10 crystal powder with the emission at 550 and 580 nm upon excitation at 340 and 390 nm, respectively; (d) UV-vis absorption spectra of DCF-10 and LCF-10 crystal powder; (e) CD signals of DCF-10 and LCF-10 under different rotation angles of the KBr pellet; (f) CPL curves of DCF-10 and LCF-10 upon excitation at 340 nm and 390 nm, respectively; (g) the dissymmetrical factor (*g*_lum_) of DCF-10 and LCF-10 under different excitation wavelengths of 340 and 390 nm, respectively.

Impressively, DCF-10 and LCF-10 crystals showed evidently dynamic long-lived luminescence behaviors under ambient conditions. The DCF-10 sample exhibits excitation-dependent phosphorescence. To further check the multiple afterglow properties, the steady-state photoluminescence spectra were recorded. As shown in Fig. 3a, the room-temperature phosphorescence spectra exhibited a remarkable red shift, and the phosphorescence peaks shifted from green to orange-red with various excitation wavelengths from 290 to 420 nm. Specifically, with a delay time of 0.1 ms, upon excitation at 340 nm, DCF-10 crystalline powder exhibited multiple phosphorescence emission peaks at 525 and 565 nm, respectively, while the maximum emission wavelength is approximately at 600 nm as the excitation wavelength turned to 390 nm. Interestingly, a new emission peak at 395 nm appears in the stead-state photoluminescence spectra upon 340 nm excitation, and this emission band reduced and further disappear in the delayed spectra with increasing the delay time 0.2 ms (Fig. S18†). This band completely overlaps with the prompt fluorescence band with a lifetime of 115.74 μs (Fig. 4b). Given this, the photoluminescence spectra were measured at varied temperatures from 77 to 393 K (Fig. S19†); the emission intensity gradually decreases with increasing temperature, as a result, it can exclude the thermally activated delayed fluorescence (TADF), and the delayed fluorescence probably arose from triplet–triplet annihilation (TTA).^40^ The Commission International de l'Eclairage (CIE) coordinate diagram also shows that the color of the phosphorescence displayed a nonlinear trend and gradually changed from green to yellow to orange, indicating that DCF-10 and LCF-10 crystals indeed have the excitation wavelength-responsive RTP (Fig. 3c). The excitation–phosphorescence mapping clearly revealed the existence of multiple excitation centers (Fig. 3b).^41,42^ Increasing the wavelength of excitation from 310 to 420 nm led to the dynamic changes of emission peaks, including the relative intensity and wavelength range, further resulting in the formation of the excitation-dependent colorful RTP. Interestingly, the CIE coordinates of photoluminescence containing fluorescence and phosphorescence of DCF-10 are (0.32, 0.35), which is very close to those of the ideal white light (0.33, 0.33), suggesting that DCF-10 can act as a single-phased white-light emission phosphor (Fig. S20†).

The RTP lifetime at 550 is 201.36 ms under ambient conditions, and the afterglow bands at 580 nm with the lifetimes of 110.23 ms can be detected at an excitation wavelength of 390 nm. Generally, the longest phosphorescence lifetimes were obtained exactly at 550 nm, the central location of the ideal green light. The lifetimes become short whether in the long wavelengths or in the short wavelengths (Fig. S21†). The maximum phosphorescence quantum efficiency of DCF-10 is about 9.88% (Fig. S22†). Notably, from the point of molecule packing, Trzpy molecules in DCF-10 show a relatively loose arrangement rather than the H-aggregation patterns in pure Trzpy crystals. The phosphorescence behaviours of DCF-10 could be largely attributed to the coordination effect,; the molecules can be well immobilized into the framework *via* the coordination lattice, thus, the molecular movement could be substantially restrained, and it can well stabilize the triplet excited state and can suppress the non-radiative transitions from the triplet excited states to ground state.^43^

The optical activity of DCF-10 and LCF-10 (well-ground powder) was evaluated by the solid-state CD and CPL technologies. Mirror-imaged Cotton bands in the range of 210–600 nm were detected in agreement with the solid-state absorption of DCF-10 and LCF-10 (Fig. 4d); the negative CD signal belongs to LCF-10, and the positive CD signal belongs to DCF-10. Moreover, almost no change was observed in the CD spectra with rotation of the KBr pellet by 90° intervals (Fig. 4e); moreover, the particle size of the crystal powder was basically well distributed in the range of 350–550 nm. These results mean that it can eliminate the impact of birefringence on CD occurrence, and the CD signals of the crystal powder are only ascribed to the well-ordered chiral aggregation, just as the previously reported chiral liquid crystal film or crystal samples.^44,45^ Importantly, it can also confirm the correction of CPL measurement below. Furthermore, this pair of homochiral coordination polymers was also confirmed by the excitation-dependent afterglow emissions. Intense mirror-imaged CPL signals at about 428 nm were observed under excitation at 340 nm. Notably, the maximum CPL signal redshifts to 550 nm by increasing the excitation wavelength to 390 nm, attributed to phosphorescence emission. We further tried to obtain the more longer emission wavelength of CPL over 600 nm by using the more longer excitation wavelength; nevertheless, no CPL signals were detected, mainly due to the fairly weak phosphorescence emission of these two compounds over 600 nm. Considering that this pair of homochiral coordination polymers has the same chiral space group on the whole, mirror-symmetry signals of DCF-10 and LCF-10 explicitly demonstrate that the directions of chiral helical chains [Zn(d-ala)]_*n*_ and [Zn(Trzpy)]_*n*_ can act as handed-selective fluorescence sieves to generate CPL, owing to the matching extent of emission of Trzpy with chiral stacking and the selective Bragg reflection behaviors of the helical environment.^46^ The level of CPL can be evaluated from the luminescence dissymmetry factor (*g*_lum_), which is defined as *g*_lum_ = 2 × (*I*_L_ − *I*_R_)/(*I*_L_ + *I*_R_), where *I*_L_ and *I*_R_ are the intensities of left- and right-handed CPL, respectively.^47^ The CPL *g*_lum_ was basically constant with the emission wavelength, and the calculated *g*_lum_ values of DCF-10 and LCF-10 are about 1.8 × 10^−3^ and 2.4 × 10^−3^ by exciting at 340 nm (Fig. 4f). Besides, it can be observed that the intense CPL signals appear in the region of fluorescence, maybe it can be reasonably explained that the TTA process of Trzpy molecules could promote fluorescence and amplify the magnetic dipole transition moment, generating a positive effect to improve the *g*_lum_ of circularly polarized emission.^48^ To the best of our knowledge, the single-phased self-assembly system featuring optical activity and the dynamic CPL properties had not been reported in the field of coordination polymers.^49^

### Anti-counterfeiting

**Fig. 5:**
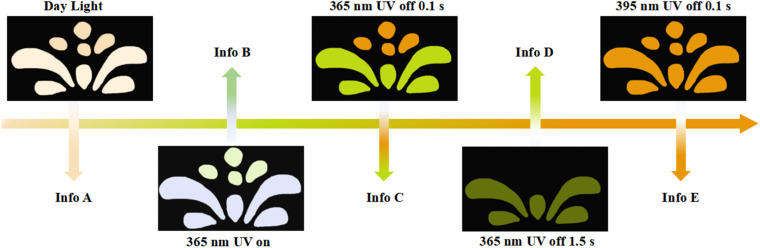
Demonstration of LCF-10 for multicolour anti-counterfeiting, the flower consists of Trzpy and DCF-10, the pistils were made of Trzpy powder, and the petals were made of LCF-10 powder. Info A: the photograph of the flower model under natural light; Info B: the flower model under a 365 nm irradiation source; Info C: the flower model after removal of the 365 nm irradiation source with a delayed time of 0.1 s; Info D: the flower model after removal of the 365 nm irradiation source with a delayed time of 1.5 s; Info E: the flower model after removal of the 395 nm irradiation source with a delayed time of 0.1 s.

The multicolor-tunable long-lived room-temperature phosphorescence of LCD-10 shows potential applications for multiple anti-counterfeiting. As shown in Fig. 5, the flower pattern was fabricated by grinding powder of Trzpy and LCF-10, and the pistils and petals were made of Trzpy powder and LCF-10 powder, respectively. Under 365 UV light, the flower showed two kinds of colors: the white color petals could be observed under excitation at 365 nm, interestingly, the color of pistils and petals changed into orange and green after stopping the 365 nm ultraviolet source of 0.1 s, and then, the pistils vanished with the delay time of 1.5 s. Furthermore, the total flower pattern exhibited an orange colour after turning off the excited light of 395 nm. Besides, the color switch is reversible under different excitation wavelengths. The time- and excitation wavelength-dependent multiple emission endowed LCF-10 with a huge advantage for multi-level encryption.

## Conclusion

In summary, we designed a nitrogen-enriched small molecule Trzpy that could achieve long-lived organic RTP. Making full use of the advantages of reticular chemistry, two enantiomers DCF-10 and LCF-10 were prepared through the chirality-induced precise assembly of Trzpy and d/l-alanine. These two homochiral coordination polymers show remarkable excitation-dependent room-temperature afterglow, and the color of the room-temperature afterglow covers an extremely broad region and switched from blue-green to green to orange by tuning the excitation wavelength from 290 to 420 nm. Significantly, circularly polarized luminescence including fluorescence and phosphorescence was realized from solid powder of two homochiral coordination polymers. Extraordinarily, excitation-responsive CPL can be also achieved. The excitation-responsive phosphorescence mechanism was deeply investigated by combining the experimental and theoretical calculations, revealing that the phosphorescence emission behaviors were dominated by the molecular packing formations and the aggregate states. Monomer, dimer, and tetranuclear clusters, even other clusters with a higher polymerization degree in the Trzpy crystal with different Δ*E*_st,_ provide multiple emission centers. Furthermore, these coordination compounds featuring multicolor-tunable optical properties exhibit huge advantages for anti-counterfeiting. The work paves a new way for the development of dynamic response room-temperature delayed luminescence and circularly polarized luminescence, and expands the application for multi-information encryption.

## Data availability

All necessary information is included in the ESI.†

## Author contributions

H. Liu and D. Ren contributed equally to this work. H. Fu and L. Ma designed and supervised the project. H. Liu, D. Ren, P. Gao, and K. Zhang performed the experiments and analyzed the preliminary results. All authors analyzed the final results. The manuscript was written by H. Liu, D. Ren, and Y. Pan.

## Conflicts of interest

The authors declare no competing financial interest.

## Supplementary Material

SC-013-D2SC05353E-s001

SC-013-D2SC05353E-s002
