# Supplementary material for: Multicolor-tunable room-temperature afterglow and circularly polarized luminescence in chirality-induced coordination assemblies
Source: Chem Sci. 2022 Nov 7;13(46):13922–9. doi: 10.1039/d2sc05353e (PMC9710219; doi:10.1039/d2sc05353e)
Supplement: SC-013-D2SC05353E-s001 [file SC-013-D2SC05353E-s001.pdf]

Electronic Supplementary Material (ESI) for Chemical Science.
This journal is © The Royal Society of Chemistry 2022

Supporting Information

For

Multicolor-tunable room temperature afterglow and circularly polarized luminescence in chirality-induced coordination assemblies

Hui Liu,[a,b] Dan-Dan Ren,[a,b] Peng-Fu Gao,[a,c] Kun Zhang,[a,b] Ya-Pan Wu,[b] Hong-Ru Fu,[*a,b] Lu-Fang Ma[*]

[a]College of Chemistry and Chemical Engineering, Henan Province Function-oriented Porous Materials Key Laboratory, Luoyang Normal University, Luoyang 471934, P. R. China.
[b]College of Materials and Chemical Engineering, China Three Gorges University, Yichang 443002 China.
[c]College of Chemistry and Chemical Engineering, Henan Polytechnic University, Jiaozuo 454003, China.
H. L. and D. D. Ren contributed equally to this work.
* Correspondance e-mail : *hongrufu2015@163.com*, *mazhuxp@126.com*

TABLE OF CONTENTS

Experimental Procedures

S1. Methods and Materials

S2. The synthesis of DCF-10 and LCF-10

S3. The method of recrystallization of Trzpy

S4. X-ray Collection and Structure Determination

S5. Computational details

Results and Discussion

S6. The UV of Trzpy and PXRD and TG data of DCF-10 and LCF-10

S7. Liquid UV absorption Spectra of TrzPy from 1 × 10-5 to 1 × 10-2 in five different solutions

S8. The Luminous Properties of D/LCF-10 and TrzPy

S9. Crystallographic data

Experiments and Method

S1. Methods and Materials

Powder X-ray diffraction (PXRD) patterns were collected on a Bruker D8 ADVANCE diffractometer with Cu Kα (λ = 1.5418 Å) radiation. Measurements were operated power was 40 KV, 30 mA, and a 2θ range of 5−50° at room temperature with a counting time of 0.2 s/ step. Single-crystal X-ray diffraction data were obtained from Bruker SMART APEX CCD diffractometer equipped with Mo-Kα radiation (λ = 0.71073 Å) at room temperature. The crystal structures were solved by OLEX2. Thermogravimetric analysis (TGA) was performed on a PerkinElmer TG-7 analyzer heated from 30 to 800 °C at the atmosphere with a ramp rate of 5 °C/min. Ultraviolet-visible (UV-vis) absorption spectra were collected on a PerkinElmer Lambda spectrophotometer. The fluorescence and phosphorescence spectra were conducted on Edinburgh FLS1000 with xenon arc lamp and nanosecond flash-lamp. The intrinsic circularly polarized luminescence (CPL) spectra were measured at the scanning speed rate of 100 nm/min on JASCO CPL-300 spectrometer. The circular dichroism (CD) spectra were measured from 200-800 nm range using JASCO J-810 circular dichroism spectrometer.

All reagents and solvents were purchased commercially. Ethyl alcohol (EtOH) and N, N-dimethylformamide (DMF) (China National Pharmaceutical Group Co., Ltd.). Triazolo[4,5-b]pyridine (TCI). The singlet oxygen detection (SOSG) is purchased on the official website of Thermo Fisher Scientific.

S2. The synthesis of DCF-10 and LCF-10

Synthesis of Zn(Trzpy)(D-ala) (DCF-10): $Zn(OAC)_2{\cdot}2H_2O$ (0.066 g, 0.30 mmol), 1H-1,2,3-Triazolo[4,5-b]pyridine (Trzpy, 0.036 g, 0.30 mmol), D-Alanine (D-ala, 0.027 g, 0.30 mmol) were dissolved in the mixed solvent of 7 mL EtOH/DMF/H_2O (v/v/v : 4//2/1 mL). The final mixture was sealed into a Teflon-lined stainless steel vessel (20 mL), heated at 100 ^{o}C for 2 days, and then cooled down to room temperature. Brown crystals were obtained and washed with DMF and ethanol several times, then dried at room temperature. Finally, crystals were collected in about 75% yield based on Trzpy. Anal. Calcd for $C_8H_8N_5O_2Zn$: Calcd. C, 35.35; H, 2.95; N, 25.83; Found C, 35.27; H, 3.00; N, 25.91. LCF-10 crystals were synthesized only by using L-alanine to replace D-alanine under the

same condition with a 71% yield.

In fact, we are hungry for a series of chiral coordination polymers to be synthesized via the induction of amino acids, unfortunately, only alanine acids were introduced into chiral coordination polymers. We will continue to prepare chiral frameworks with the incorporation of other amino acids.

S3. The method of recrystallization of Trzpy

1H-1,2,3-Triazolo[4,5-b]pyridine 30 mg was dissolved in 15 ml deionized water in a glass vial, heated at 100 ℃ for 2 hours. Then the vial was kept in the dark at room temperature for slow evaporation of solvent. Well-formed crystals suitable for single crystal X-ray diffraction measurement appeared within approximately four days. The product was isolated prior to total evaporation of the solvent to guarantee the crystal purity.

S4. X-ray Collection and Structure Determination

The diffraction data were measured at 293(2) K under nitrogen flowing with Rigaku supernova CCD system equipped withconfocal monochromated Mo-Kα radiation (λ = 0.71073 Å). All structures were solved by a direct method and refined by using Olex2.0,[1] through the assistance of SHELXTL program.[2] The hydrogen atoms were processed in calculated model directly on the organic ligands and the coordinated solvent molecules. The hydrogen atoms, which was distributed on the coordinated water molecules, were not added due to the lack of hydrogen bond acceptors. The free solvent molecules and other unambiguously residues were all removed through a SQUEEZE operation by PLATON.[3] Crystallographic data for DCF-10 and LCF-10 are listed in Table S1 and S2.

S5. Computational details

The computational models were built from the crystal structure. The ground state (S_0) and excited state geometries of Trzpy are fully optimized by density functional theory (DFT) method and time-

dependent density functional theory (TD-DFT) method, respectively. All these calculations are performed by Gaussian 16 package with B3LYP functional and 6-31G(d) basis set.[4]

Results and Discussion

S6. The UV of Trzpy and PXRD and TGA curves of DCF-10 and LCF-10

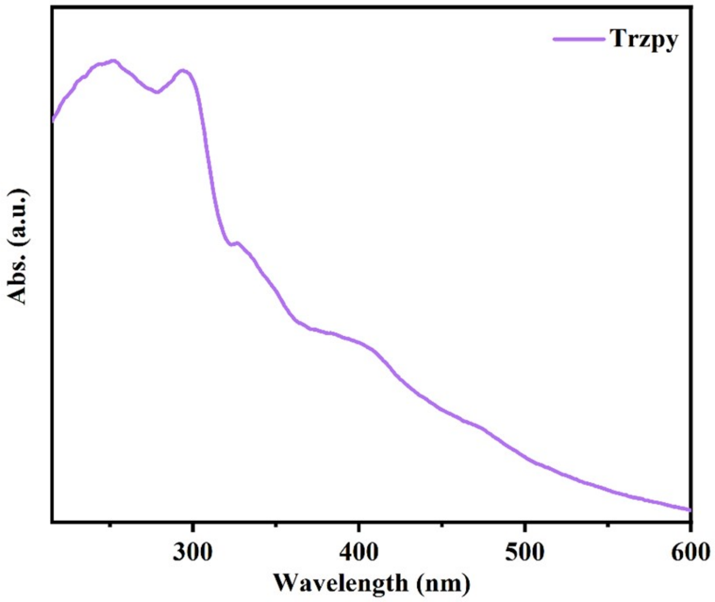

Fig. S1 UV-vis adsorption spectra of Trzpy powder.

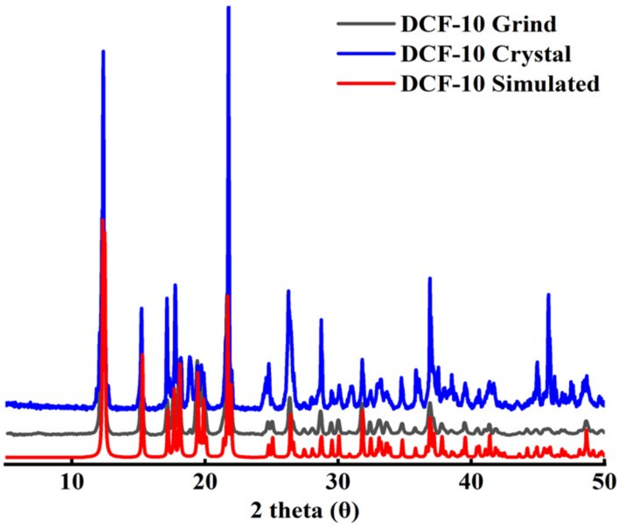

Fig. S2 PXRD patterns of DCF-10.

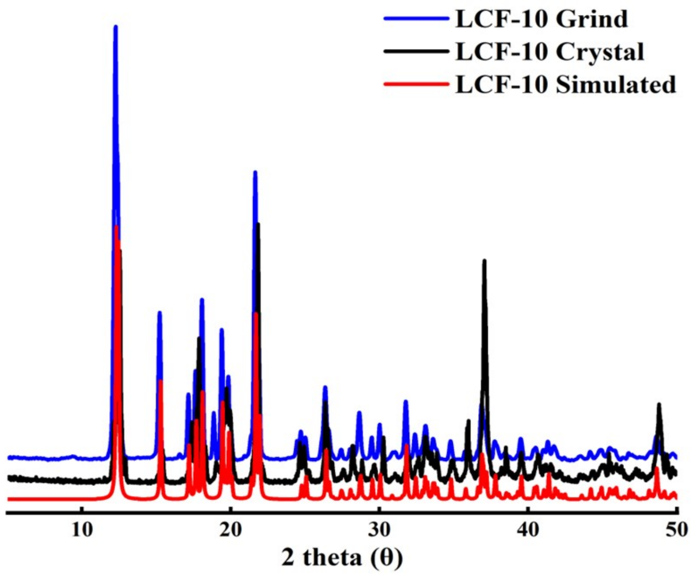

Fig. S3 PXRD patterns of LCF-10.

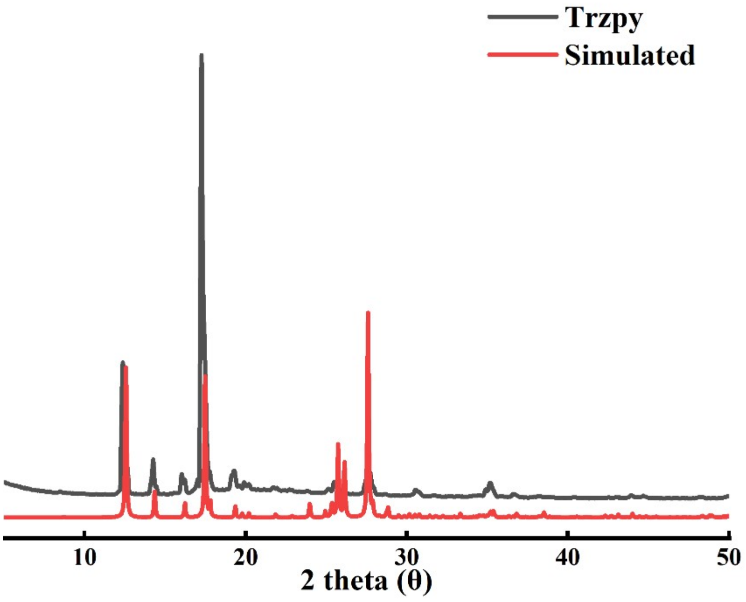

Fig. S4 PXRD patterns of Trzpy sample.

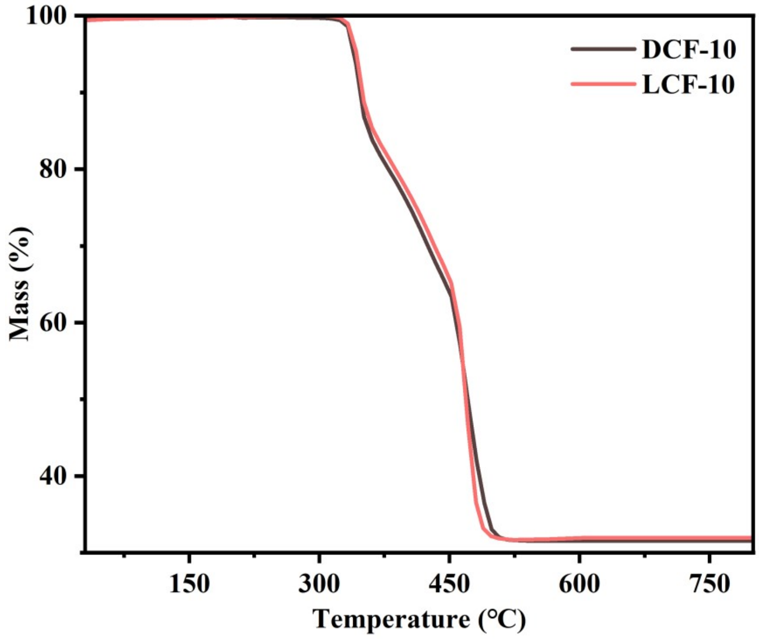

Fig. S5 TGA curves of DCF 10 and LCF 10 under air atmosphere.

S7. Liquid UV absorption Spectra of Trzpy from 1 × 10^{-5} to 1 × 10^{-2} in five different solutions.

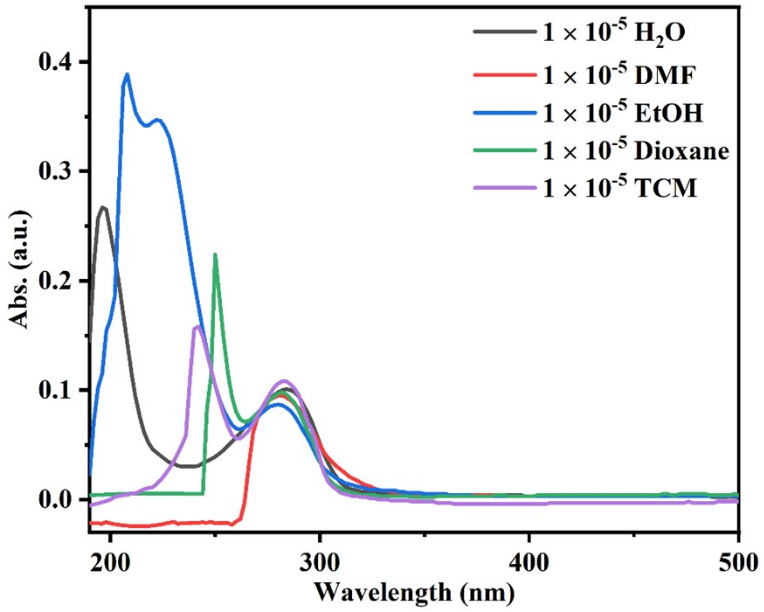

Fig. S6 Liquid UV absorption of Trzpy with concentration of 1×10^{-5} in distilled water (H_2O), N, N-dimethylformamide (DMF), ethanol (EtOH), 1,4-dioxane (Dioxane), trichloromethane (TCM).

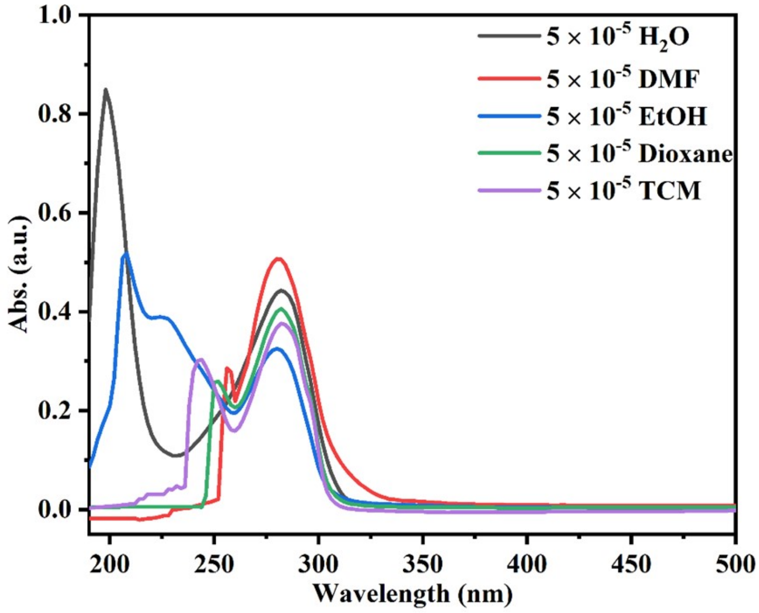

Fig. S7 Liquid UV absorption of Trzpy with concentration of 5×10^{-5} in distilled water (H_2O), N, N-dimethylformamide (DMF), ethanol (EtOH), 1,4-dioxane (Dioxane), trichloromethane (TCM).

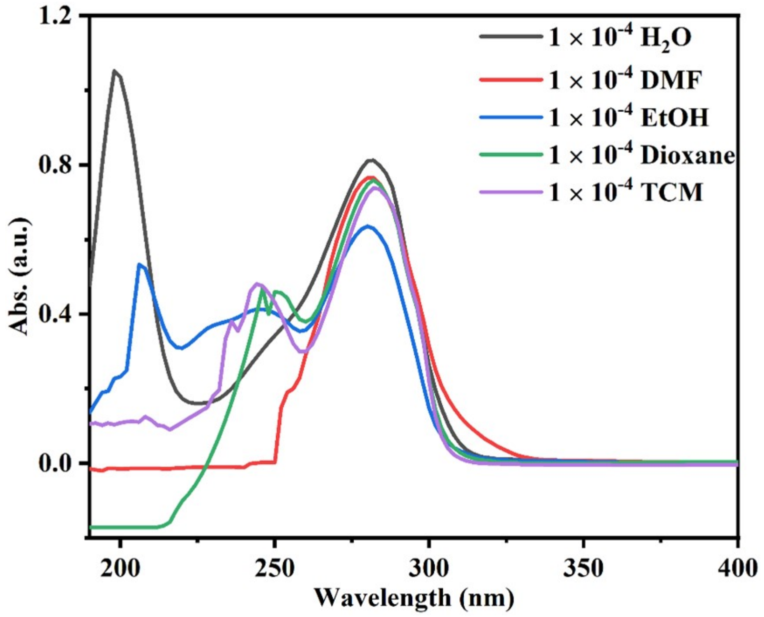

Fig. S8 Liquid UV absorption of Trzpy with concentration of 1×10^{-4} in distilled water (H_2O), N, N-dimethylformamide (DMF), ethanol (EtOH), 1,4-dioxane (Dioxane), trichloromethane (TCM).

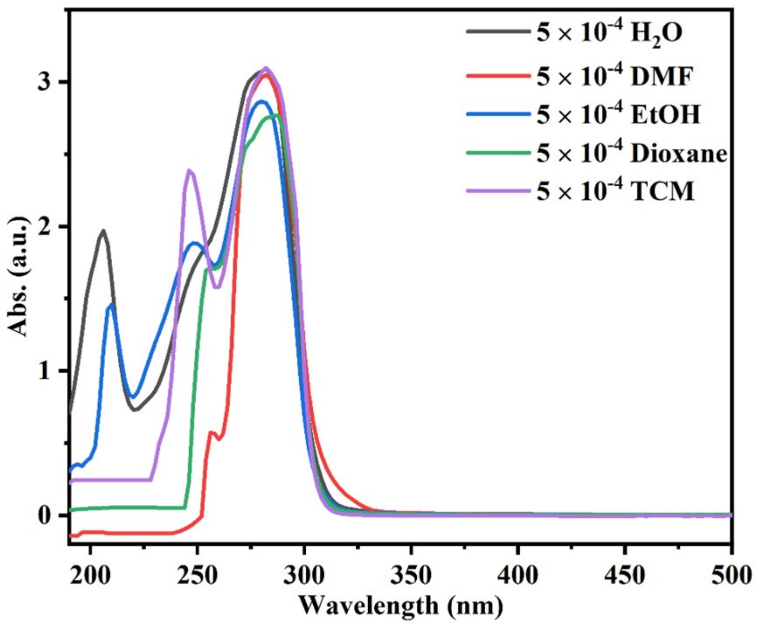

Fig. S9 Liquid UV absorption of Trzpy with concentration of 5×10^{-4} in distilled water (H_2O), N, N-dimethylformamide (DMF), ethanol (EtOH), 1,4-dioxane (Dioxane), trichloromethane (TCM).

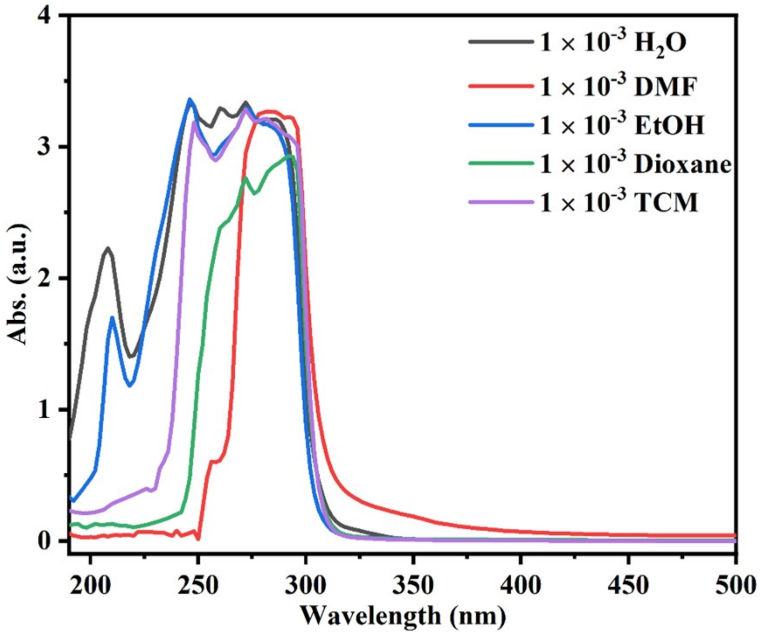

Fig. S10 Liquid UV absorption of Trzpy with concentration of 1×10^{-3} in distilled water (H_2O), N, N-dimethylformamide (DMF), ethanol (EtOH), 1,4-dioxane (Dioxane), trichloromethane (TCM).

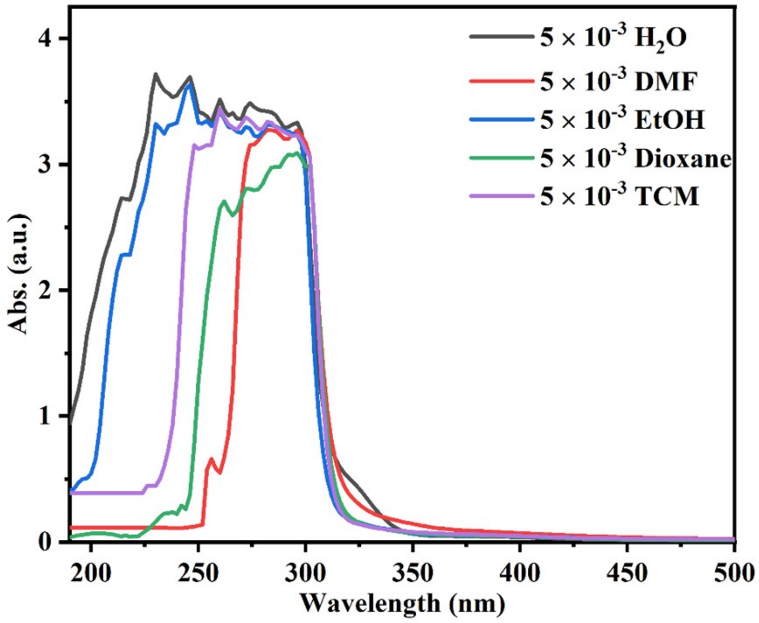

Fig. S11 Liquid UV absorption of Trzpy with concentration of 5×10^{-3} in distilled water (H_2O), N, N-dimethylformamide (DMF), ethanol (EtOH), 1,4-dioxane (Dioxane), trichloromethane (TCM).

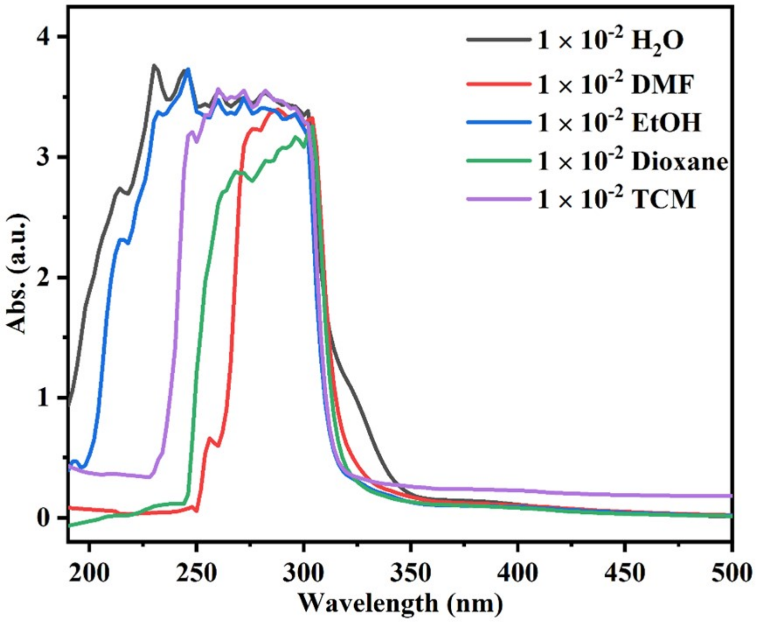

Fig. S12 Liquid UV absorption of Trzpy with concentration of 1×10^{-2} in distilled water (H_2O), N, N-dimethylformamide (DMF), ethanol (EtOH), 1,4-dioxane (Dioxane), trichloromethane (TCM).

S8. The Luminous Properties of D/LCF-10 and TrzPy

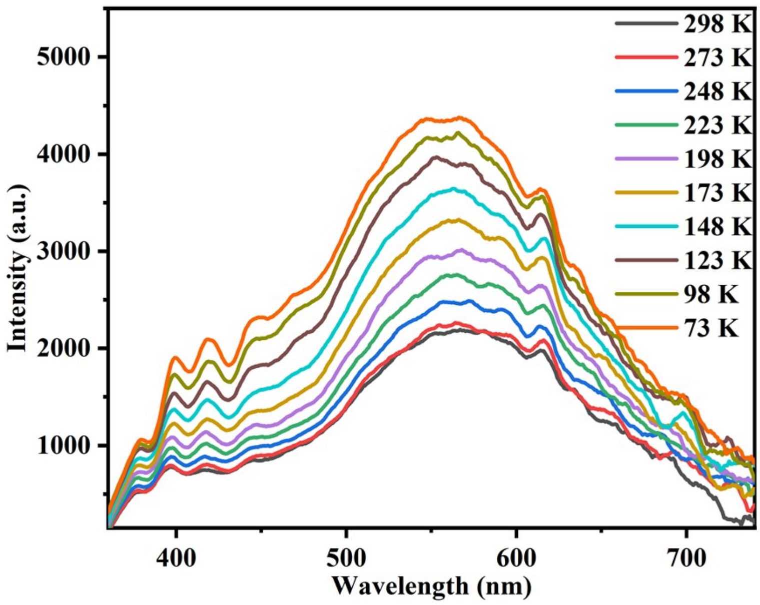

Fig. S13 The photoluminescence spectrum of Trzpy powder under different temperatures from 77 to 298 K, the emission intensity gradually decreased with the increasing temperature.

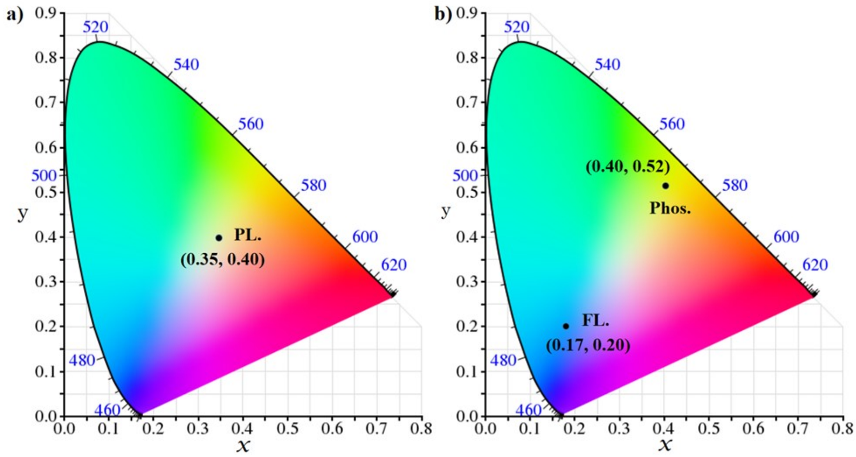

Fig. S14 Calculated photoluminescence (PL.), fluorescence (FL.) and phosphorescence (Phos.) color coordinates of the solid Trzpy by the CIE 1931 chromaticity diagram.

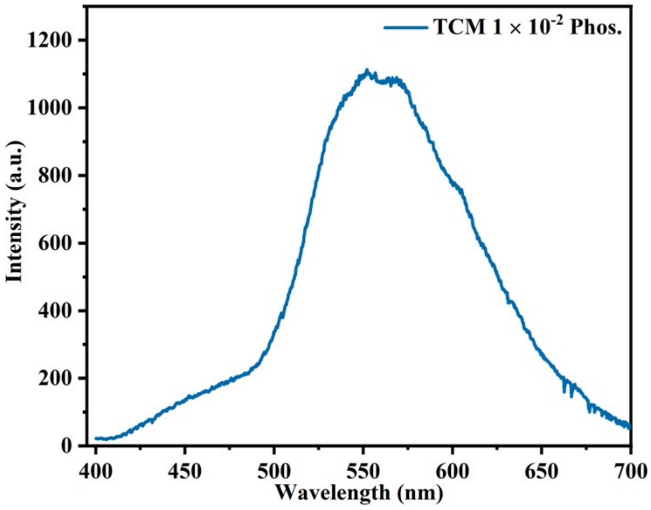

Fig. S15 Phosphorescence spectrum of Trzpy with a concentration of 1 × 10^{-2} in trichloromethane (TCM).

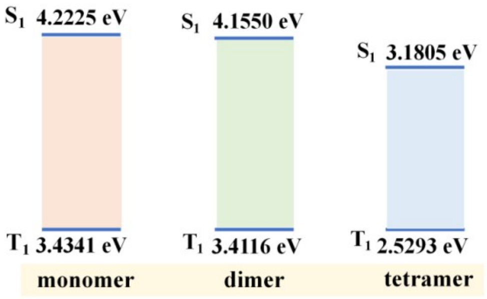

Fig.S16 Singlet-triplet energy of Trzpy molecule calculated from the single crystal.

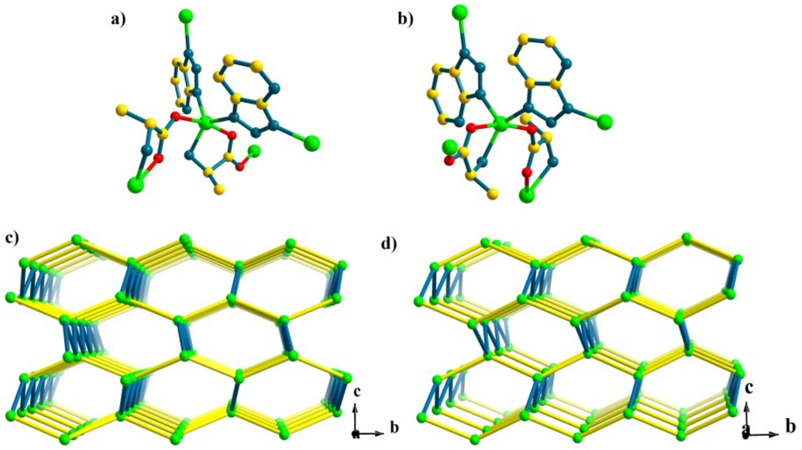

Fig. S17 (a) Coordination environment of Zn^{2+} ion in DCF-10; (b) Coordination environment of Zn^{2+} ion in LCF-10; (c) Dia-type topology of DCF-10; (d) Dia-type topology of LCF-10.

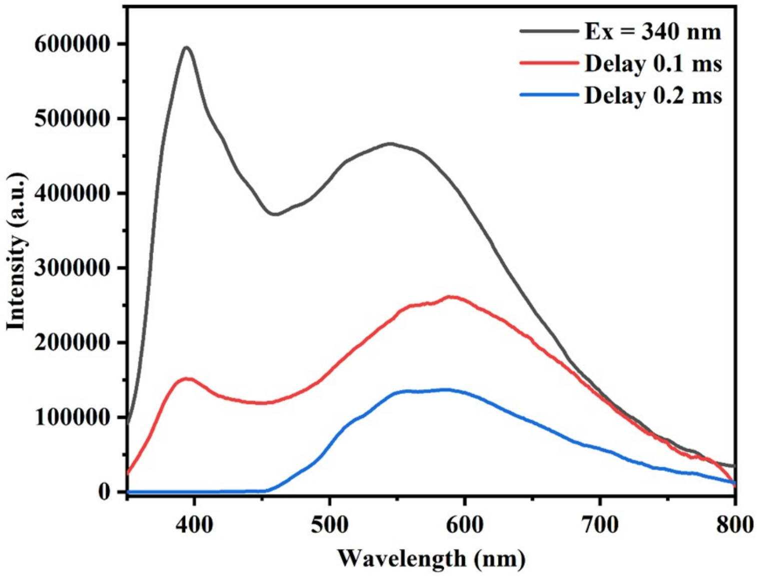

Fig. S18 Delayed photoluminescence emission spectra of DCF-10, indicating the delayed luminescence has two peaks at 400 and 595 nm or so.

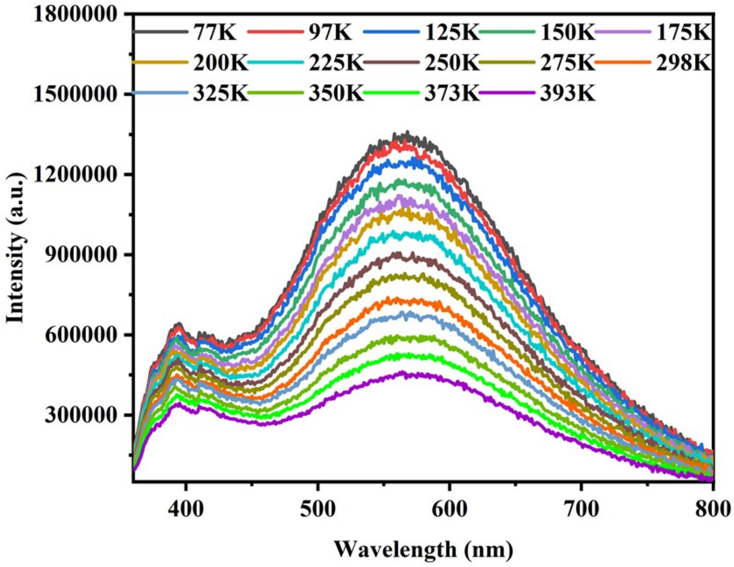

Fig. S19 The photoluminescence spectrum of DCF-10 under the varied temperature from 77 to 393 K.

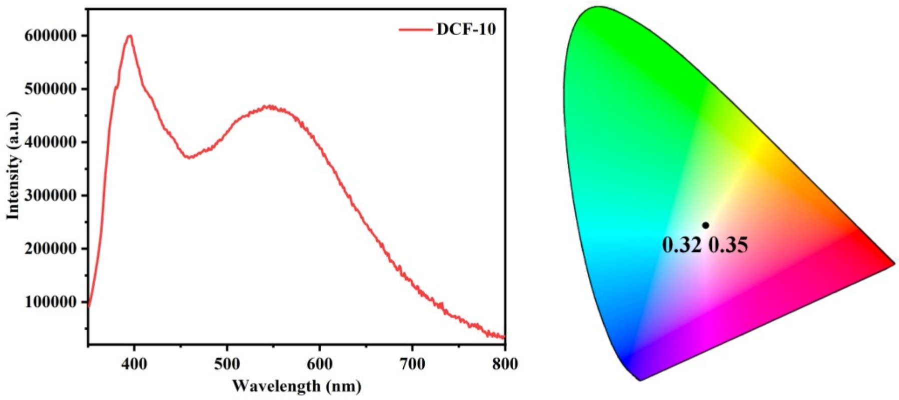

Fig. S20 The photoluminescence spectra of DCF-10, and corresponding CIE coordinate diagram.

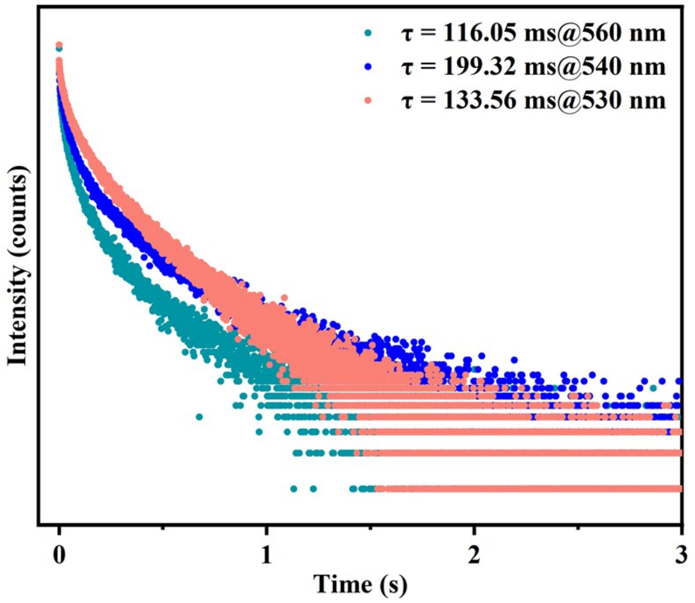

Fig. S21 Phosphorescence decay curves of DCF-10 with the emission wavelengths at 530, 540 and 560 nm, respectively.

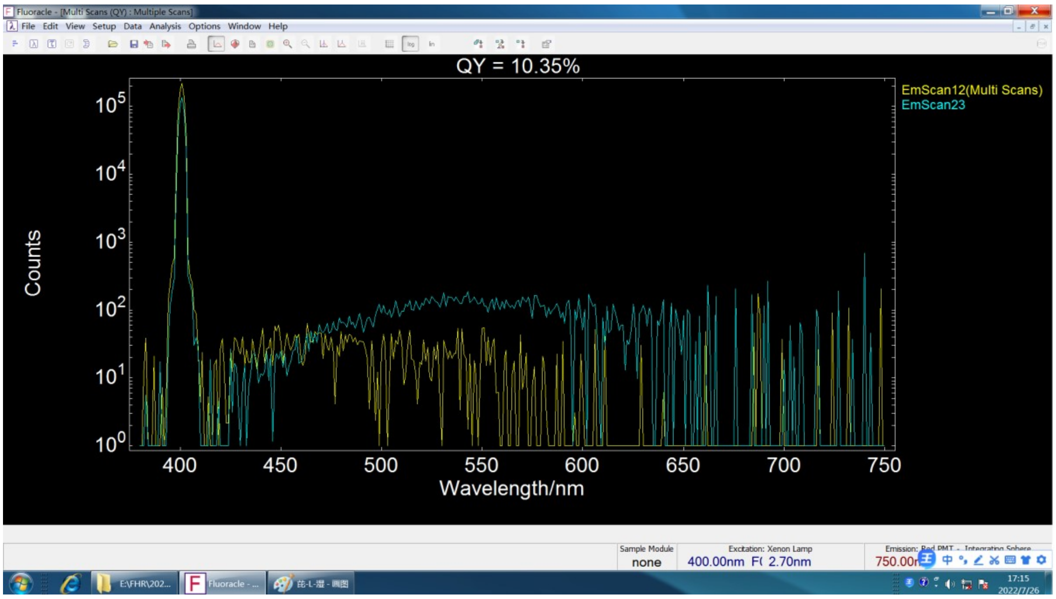

Fig. S22 Phosphorescence quantum yield of LCF-10.

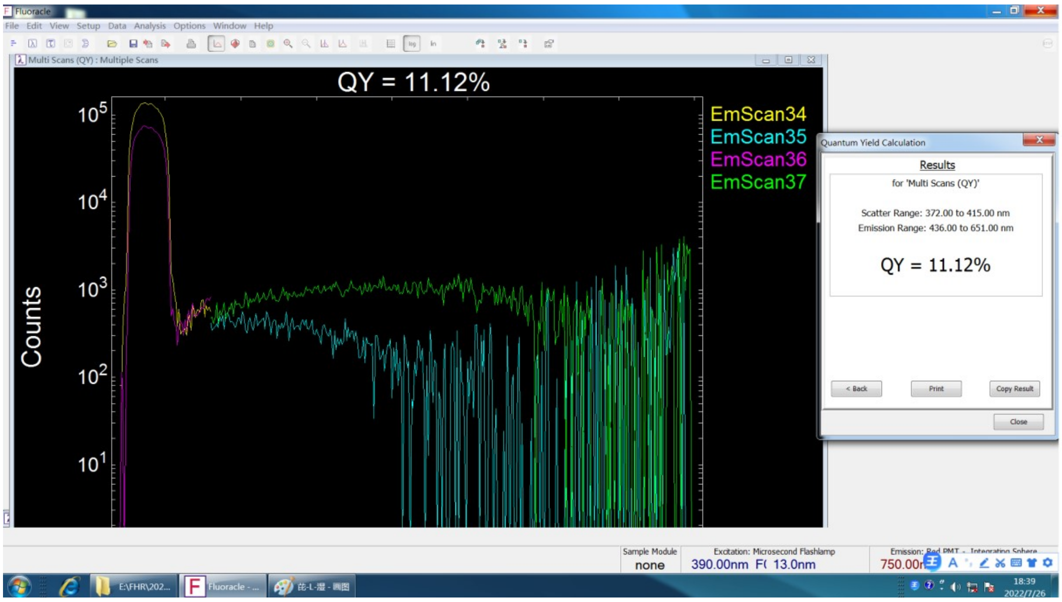

Fig. S23 Phosphorescence quantum yield of DCF-10.

S9. Crystallographic data.

Table S1. Single crystal diffraction data of Trzpy.

Identification code	Trzpy
Empirical formula	$C_{10}H_8N_8$
Number of CCDC	2195443
Formula weight	240.24
Temperature/K	293(2)
Crystal system	triclinic
Space group	*P*-1
a / Å	3.7561(4)
b / Å	10.2354(13)
c / Å	14.2732(16)
α / °	97.137(10)
β / °	96.169(9)
γ / °	92.497(11)
Volume/ Å^3	540.49(11)
Z	2
ρ_{calc}g/cm^3	1.476
μ / mm^{-1}	0.102
F(000)	248.0
Crystal size/ mm^3	0.2 × 0.15 × 0.10
Radiation	MoKα (λ = 0.71073)
2θ range for data collection/°	6.606 to 58.416
Index ranges	$-4 \le h \le 4$, $-13 \le k \le 13$, $-18 \le l \le 18$
Reflections collected	4445
Independent reflections	2438 [R_{int} = 0.0337 R_{sigma} = 0.0882]
Goodness-of-fit on F^2	1.095
R_1 [I>2σ (I)]	R1 = 0.0746, wR2 = 0.1374
wR_2 (all data)	R1 = 0.1484, wR2 = 0.1705

Table S2. Single crystal diffraction data DCF-10 and LCF-10.

Identification code	DCF-10	LCF-10
Empirical formula	$C_8H_9N_5O_2Zn$	$C_8H_9N_5O_2Zn$
Number of CCDC	2195444	2195445
Formula weight	272.59	272.57
Temperature/K	293(2)	293(2)
Crystal system	orthorhombic	orthorhombic
Space group	$P2_12_12_1$	$P2_12_12_1$
a / Å	9.7893(5)	9.7703(9)
b / Å	10.0252(7)	9.9768(10)
c / Å	10.2988(6)	10.6503(9)
α / °	90	90
β / °	90	90
γ / °	90	90
Volume/ $Å^3$	1010.72(11)	1038.15(17)
Z	4	4
ρ_{calc}g/cm^3	1.791	1.744
μ / mm^{-1}	2.422	2.358
F(000)	552.0	552.0
Crystal size/ mm^3	0.11 × 0.11 × 0.10	0.2 × 0.2 × 0.2
Radiation	Mo-Kα (λ = 0.71073)	Mo-Kα (λ = 0.71073)
2θ range for data collection/°	7.036 to 58.252	6.98 to 58.584
Index ranges	$-13 \le h \le 6$, $-7 \le k \le 12$, $-12 \le l \le 13$	$-6 \le h \le 13$, $-6 \le k \le 12$, $-13 \le l \le 12$
Reflections collected	3248	3279
Independent reflections	2136 [R_{int} = 0.0218 R_{sigma} = 0.0488]	2126 [R_{int} = 0.0242, R_{sigma} = 0.0503]
Flack	0.009(15)	0.009(15)
Goodness-of-fit on F^2	1.065	1.057
R_1 [I>2σ (I)]	R_1 = 0.0333, wR_2 = 0.0600	R_1 = 0.0348, wR_2 = 0.0652
wR_2 (all data)	R_1 = 0.0370, wR_2 = 0.0626	R_1 = 0.0379, wR_2 = 0.0676

REFERENCES

(1) Spek, A. L. Crystal structure refinement with *SHELXL*. *Acta Crystallogr., Sect. C* **2015**, 71, 3–8.

(2) Sheldrick, G. M. *SHELXT*–Integrated space-group and crystal-structure determination. *Acta Cryst.* **2015**, A71, 3−8.

(3) Spek, A. L. PLATON SQUEEZE: A Tool for the Calculation of the Disordered Solvent Contribution to the Calculated Structure Factors. *Acta Crystallogr. Sect. C: Struct. Chem.* **2015**, 71, 9−18.

(4) Frisch, M. J.; Trucks, G. W.; Schlegel, H. B.; Scuseria, G. E.; Robb, M. A.; Cheeseman, J. R.; Scalmani, G.; Barone, V.; Mennucci, B.; Petersson, G. A.; Nakatsuji, H.; Caricato, M.; Li, X.; Hratchian, H. P.; Izmaylov, A. F.; Bloino, J.; Zheng, G.; Sonnenberg, J. L.; Hada, M.; Ehara, M.; Toyota, K.; Fukuda, R.; Hasegawa, J.; Ishida, M.; Nakajima, T.; Honda, Y.; Kitao, O.; Nakai, H.; Vreven, T.; Montgomery Jr., J. A.; Peralta, J. E.; Ogliaro, F.; Bearpark, M. J.; Heyd, J.; Brothers, E. N.; Kudin, K. N.; Staroverov, V. N.; Kobayashi, R.; Normand, J.; Raghavachari, K.; Rendell, A. P.; Burant, J. C.; Iyengar, S. S.; Tomasi, J.; Cossi, M.; Rega, N.; Millam, N. J.; Klene, M.; Knox, J. E.; Cross, J. B.; Bakken, V.; Adamo, C.; Jaramillo, J.; Gomperts, R.; Stratmann, R. E.; Yazyev, O.; Austin, A. J.; Cammi, R.; Pomelli, C.; Ochterski, J. W.; Martin, R. L.; Morokuma, K.; Zakrzewski, V. G.; Voth, G. A.; Salvador, P.; Dannenberg, J. J.; Dapprich, S.; Daniels, A. D.; Farkas, Foresman, J. B.; Ortiz, J. V.; Cioslowski, J.; Fox, D. J. Gaussian 09, Revision D.01; Gaussian, Inc.: Wallingford, CT, **2013**.
